# Suppression of Nrf2 Activity by Chestnut Leaf Extract Increases Chemosensitivity of Breast Cancer Stem Cells to Paclitaxel

**DOI:** 10.3390/nu9070760

**Published:** 2017-07-18

**Authors:** Yaejin Woo, Jisun Oh, Jong-Sang Kim

**Affiliations:** 1 School of Food Science and Biotechnology (BK21 Plus), Kyungpook National University, Daegu 41566, Korea; yuwoo4235@naver.com; 2 Institute of Agricultural Science & Technology, Kyungpook National University, Daegu 41566, Korea

**Keywords:** breast cancer, cancer stem cell, Nrf2, drug resistance, *Castanea crenata*

## Abstract

Due to metastatic potential and drug resistance, cancer stem cells (CSCs) have become a critical target for the development of chemotherapeutic agents. Recent studies showed that CSCs highly express NF-E2-related factor 2 (Nrf2)-mediated antioxidant enzymes and thereby retain relatively low levels of reactive oxygen species (ROS). Since anticancer agents usually utilize ROS as an arsenal for killing cancer cells, we hypothesized that inhibition of Nrf2 activity could increase the sensitivity of CSCs to anticancer drugs, and thus enhancing their therapeutic efficacy. We found that MCF-7-derived CSCs with a CD44^high^/CD24^low^ phenotype formed mammospheres and highly expressed Nrf2 compared to the adherent parental MCF-7 cells. In a separate experiment, we screened 89 different edible plant extracts for inhibitory activity against the Nrf2 signaling pathway by using an antioxidant response element (ARE)-luciferase assay system. Among those extracts, *Castanea crenata* (chestnut) leaf extract significantly decreased the nuclear translocation of Nrf2 and protein expression of antioxidant enzymes in MCF-7-derived CSCs. The combined treatment of the CSCs with chestnut leaf extract and paclitaxel resulted in more effective cell death than the treatment with paclitaxel alone. These findings suggest that the chestnut leaf extract or its constituents could increase the susceptibility of breast CSCs to an anticancer drug, paclitaxel, through inhibition of the Nrf2 signaling pathway, and could be utilized as an adjuvant for chemotherapy.

## 1. Introduction

The cells within a tumor are heterogeneous in tumorigenic potential. It is believed that a single clone within the tumor hierarchically diversifies into multiple subclones and the unique subset of tumor cells, called cancer stem cells (CSCs), is capable of self-renewing unlimitedly, differentiating into the tumor progeny, and reinitiating the tumor [1,2,3]. Breast CSCs possessing these capabilities are reported to be the seed of breast cancer in humans and animal models [4,5]; the cell population is mainly positive for cluster of differentiation (CD) 44 (CD44) and negative for CD24 [6,7,8] and generally cultivated as mammospheres [6,7,8,9]. Considering the tumorigenic potential of CSCs, it would be effective to target CSCs as therapeutic or preventive strategies for cancer treatment. 

The CSCs exhibit resistance against chemotherapeutic agents through clonal expansion and asymmetric division, active DNA repair capacity, and resistance to apoptosis [10]. Recent studies have shown that CSCs maintain intracellular reactive oxygen species (ROS), byproducts of aerobic metabolism, at relatively low levels, although rapidly propagating non-stem cancer cells have increased levels of ROS compared to their normal counterparts [8,11]. Based on the evidence that the antioxidant transcription factor NF-E2-related factor 2 (Nrf2) is constitutively activated in CSCs, it is appreciated that Nrf2-mediated cytoprotective responses contribute to the resistance of CSCs to therapeutic agent-induced ROS insults, the arsenal for cancer treatment [12,13,14,15]. Breast CSCs, for instance, have low ROS levels [11,16] due to upregulation of ROS-scavenging systems, including Nrf2-mediated antioxidant signaling molecules [5,17], which contributes to paclitaxel resistance in the CSCs [4,12].

Numerous plants have been reported—with or without experimental evidence—to have anticancer effects [18]. The chemical constituents of plants, including vegetables, fruits, grains, and herbs, have been reported to possess anticancer properties [18,19,20]. Plant-derived anticancer agents can inhibit cancer cell survival and propagation by interfering with microtubule stabilization, limitation of cancer cell proliferation, or disruption of chromatin structure [19]. Intriguingly, recent studies have shown that certain plant-derived substances, such as brusatol, can sensitize cancer or cancer stem-like cells to conventional chemotherapeutic drugs by inhibiting the cytoprotective function of Nrf2. It indicates that the intracellular ROS level increases in CSCs by Nrf2 inhibitor could enhance the therapeutic efficacy of anticancer drugs [4,21,22,23]. 

As such, the present study examined the potential of chestnut leaf extract to inhibit the function of Nrf2 in breast CSCs and thereby enhance the sensitivity of CSCs to paclitaxel, a Food and Drug Administration-approved and widely used anticancer drug for breast cancer chemotherapy. In this study, we demonstrate that the chestnut leaf extract or its constituents could increase the susceptibility of breast CSCs to paclitaxel through inhibition of Nrf2 signaling pathway. 

## 2. Materials and Methods

### 2.1. Preparation of Plant Extracts

A variety of plant extracts including *Castanea crenata* (chestnut) leaf extract were obtained from the Korea Plant Extract Bank (Ochang, Chungbuk, Korea). According to the supplier, each plant was dried, ground, and extracted in methanol. The extracts were filtered, vacuum-evaporated, and lyophilized. For in vitro measurement of antioxidant activity, the extracts were dissolved in dimethyl sulfoxide (DMSO; Sigma-Aldrich, St. Louis, MO, USA) at 20 mg/mL, and used at the designated concentrations.

### 2.2. Cell Culture

MCF-7, a human breast cancer cell line and widely used for studying cellular features of CSCs, was obtained from KCLB (Korean Cell Line Bank, Seoul, Korea). Cells were cultured in a maintenance medium containing Dulbecco’s modified Eagle’s medium (DMEM), 10% fetal bovine serum (FBS), 25 mM hydroxyethyl piperazineethanesulfonic acid (HEPES), 1% minimum essential medium non-essential amino acids (NEAA), and 1% penicillin-streptomycin (all from Invitrogen/Life Technologies, Carlsbad, CA, USA). For sub-cultivation, cells were rinsed in phosphate-buffered saline (PBS; Gibco/Life Technologies, Carlsbad, CA, USA), detached using 0.05% Trypsin-ethylenediaminetetraacetic acid (EDTA) (Gibco/Life Technologies, Carlsbad, CA, USA), harvested, plated onto prepared culture dishes, and maintained in culture incubator (37 °C, 5% CO_2_, humidified).

MCF-7-derived CSCs were maintained in a CSC maintenance medium, in which the basal medium was 1:1 mixture of DMEM and Nutrient Mixture F-12 (DMEM/F-12; Invitrogen/Life Technologies), supplemented with 1X ITS universal culture supplement premix (BD Biosciences, San Jose, CA, USA), 10% KnockOut^TM^ Serum Replacement (KSR; Invitrogen/Life Technologies), 0.5% NEAA, 0.01% β-mercaptoethanol (EMD Millipore Corp., Billerica, MA, USA), 1% penicillin-streptomycin, 5 ng/mL of basic fibroblast growth factor (human recombinant bFGF; Invitrogen/Life Technologies), 5 ng/mL of epidermal growth factor (human EGF; Peprotech, Rocky Hill, NJ, USA), and 10% MEGM™ mammary epithelial cell growth medium (Lonza Group Ltd., Basel, Switzerland). 

### 2.3. Fluorescence-Activated Cell Sorting (FACS)

To sort the MCF-7-derived CSCs, FACS was performed using the BD FACSAria™ III flow cytometer (BD Biosciences) for MCF-7 cells with CD44^high^/CD24^low^ phenotype [6]. MCF-7 cells (1 × 10^6^ cells) were harvested and resuspended in 100 μL of Hank’s Balanced Salt Solution (HBSS; Gibco/Life Technologies, Carlsbad, CA, USA) containing 2% FBS. With 15 μL of each antibody (mouse anti-human CD44, fluorescein isothiocyanate (FITC)-conjugated; mouse anti-human CD24, phycoerythrin (PE)-conjugated; both were obtained from BD Biosciences) added, cells were incubated for 30 min at 4 °C in dark. After washing twice in 2% FBS/HBSS, cells were subjected to flow cytometry. CD44^high^/CD24^low^ cells (about 5% of parental MCF-7 cells) were then collected [24] and kept in the CSC maintenance medium in a formation of mammospheres until further analysis. 

### 2.4. Determination of Cytotoxicity 

To test the cytotoxicity of each extract, a cell counting kit-8 (CCK-8; Dojindo Laboratories, Kumamoto, Japan) was used as previously described [25]. Cells were treated with various concentrations of the chestnut leaf extract or an anti-cancer substance or both, paclitaxel (Taxol), followed by incubation for 48 h. The CCK-8 assay was performed as per manufacturer’s instructions. The absorbance, which is proportional to the number of living cells in each well, was measured at 450 nm using a microplate reader (Sunrise™, Tecan Group Ltd., Männedorf, Switzerland).

### 2.5. Cell Death Analysis

To determine the portion of alive or dead cells according to the designated treatment, a FITC-conjugated annexin V and red-fluorescent propidium iodide (PI)-based assay was performed (FITC Annexin V Apoptosis Detection Kit I; BD Biosciences). Cells externalizing the membrane phosphatidylserine, which was recognized by annexin V, a calcium-dependent phospholipid-binding protein, and/or cells permeable to PI were stained and identified using the FACSCalibur^TM^ flow cytometer (BD Biosciences). 

### 2.6. Measurement of Antioxidant Response Element (ARE) Activity

A human hepatoma cell line HepG2 was obtained from the Korean Cell Line Bank (KCLB; Seoul, Korea) and propagated in DMEM supplemented with 10% FBS and 1% penicillin-streptomycin (all from Invitrogen/Life Technologies). The cells were then transfected with pGL4.37[luc2P/ARE/Hygro] vector (Promega, Madison, WI, USA) using TurboFect transcription reagents (Thermo Fisher Scientific, Waltham, MA, USA). The transfectant carrying an ARE-luciferase construct was named HepG2-ARE and maintained in its growth medium containing 0.4 mM hygromycin (Sigma-Aldrich, St. Louis, MO, USA). 

To measure the transcriptional activity of Nrf2, luciferase reporter assay was conducted on HepG2-ARE cells as described by Kim et al. [26]. The cells were treated with samples for 12 h after serum starvation (0.5% FBS, 12 h). The luciferase activity, which corresponded to the ARE activity, was measured using a luciferase assay system (Promega) according to the manufacturer’s instruction. Sulforaphane (Sigma-Aldrich), an isothiocyanate, was used as an ARE activator [27,28]. Brusatol (Carbosynth Ltd., Newbury, Berks, UK), a quassinoid, was used as a specific inhibitor of the Nrf2 pathway [23]. The luminescence was detected using a TD-20/20 luminometer (Turner Designs, Sunnyvale, CA, USA), and calibrated to total protein amounts. The data were then normalized against the control values. 

### 2.7. Fractionation of Nuclear and Cytoplasmic Proteins

Cells were collected, rinsed, and then subjected to the NE-PER^®^ Nuclear and Cytoplasmic Extraction Reagents (Thermo Fisher Scientific) according to manufacturer’s instruction. Briefly, cells were lysed in cytoplasmic extraction reagent cytoplasmic extraction reagent (CER) I and CER II, and centrifuged at 10,000× *g* for 30 min at 4 °C. The supernatants were labeled as cytoplasmic fraction sample and stored at −20 °C for further analysis. The remainder was lysed in the nuclear extraction reagent (NER) and incubated for 40 min on ice. Subsequently, the lysate was centrifuged at 10,000× *g* for 1 h at 4 °C. The supernatants were labeled as nuclear fraction sample and stored at −20 °C for further analysis.

### 2.8. Western Blot Analysis

To determine nuclear and/or cytoplasmic Nrf2 levels in the cultured cells, the fractionated proteins were electrophoretically transferred to a polyvinylidene fluoride (PVDF) membrane (Merck Millipore Corp., Billerica, MA, USA). Primary antibodies used in this study were as follows: rabbit anti-Nrf2 (abcam, Cambridge, UK; Cat # ab62352), goat anti-Lamin B (Santa Cruz Biotechnology, Inc., Dallas, TX, USA; Cat # SC-6216), rabbit anti-Bcl-2-associated X protein (Bax; abcam; Cat # ab32503), rabbit anti-B-cell lymphoma 2 (Bcl-2; abcam; Cat # ab32124), rabbit anti-cytochrome C (abcam; Cat # ab133504), rabbit anti-poly (ADP-ribose) polymerase (PARP; Cell Signaling Technology, Inc., Danvers, MA, USA; Cat # 9542S), and mouse anti-β-actin (Santa Cruz Biotechnology; Cat # SC-47778). Secondary antibodies used were anti-mouse, anti-rabbit or anti-goat IgG, conjugated to horseradish peroxidase (Santa Cruz Biotechnology). Protein bands were visualized using the SuperSignal^®^ West Pico Chemiluminescent Substrate kit (Thermo Fisher Scientific) and imaged using ImageQuant LAS 4000 mini (GE Healthcare Life Sciences, Little Chalfont, UK). Densitometric analysis was performed using Image Studio^TM^ Lite software (LI-COR Corp., Lincoln, NE, USA).

### 2.9. Quantitative Polymerase Chain Reaction (qPCR) Analysis

Total RNA extracts were prepared from the harvested cells using a column-based isolation kit (RNeasy Mini Kit; Qiagen, Hilden, Germany) according to the manufacturer’s instructions. The extracted RNA was quantified on the basis of absorbance at 260 nm, and 0.5 μg of RNA was reverse-transcribed to cDNA using M-MLV Reverse Transcriptase (Thermo Fisher Scientific) with oligo(dT)12–18 primer. To analyze the relative transcript expression levels for respective genes, SYBR Green-based real-time PCR was performed using LightCycler^®^ Multiplex Masters (Roche, Basel, Switzerland) with the designated primer sets (Table 1) on LightCycler^®^ Nano Instrument (Roche). The transcript expression levels were normalized with the expression level of a gene encoding glyceraldehyde 3-phosphate dehydrogenase (*GAPDH*). 

### 2.10. Mitochondrial Membrane Potential Assay Using JC-1 Dye 

Cells were plated on a 12-mm diameter circular cover glass (Thermo Fisher Scientific) coated with poly-l-lysine (0.1% *w*/*v*; Sigma-Aldrich) and human plasma fibronectin (50 μg/mL; Thermo Fisher Scientific) at a density of 3 × 10^4^ cells per well in a 24-well plate. The day after, when the confluency reached approximately 70%, cells were treated with samples in 0.5% FBS-containing medium. After a 4-h incubation, cells were stained with JC-1 dye (2 μg/mL per well) for 20 min, and subsequently fixed with 3.7% formalin solution. After mounting, the stained cells were visualized under a fluorescence microscope (Eclipse 80i, Nikon, Tokyo, Japan).

### 2.11. Clonogenic Assay

Cells were cultured in a 6-well plate coated with Matrigel (2.5% *v*/*v* in PBS; Corning, Corning, NY, USA) in the absence or presence of the test sample or paclitaxel for 5 days. Colonies formed from the plated cells in each well were optically visualized via crystal violet staining. The scanned images of each well of the 6-well plate were analyzed using ImageJ software, 1.48v (developed at the National Institutes of Health). 

### 2.12. Statistical Analysis

The obtained data were analyzed via one-way analysis of variance and Duncan's multiple range test using the SPSS statistics 22 software (SPSS Inc., Chicago, IL, USA). The *p*-values less than 0.05 were considered significant. Statistical differences were indicated using asterisks, hashtags, or different alphabetical letters. 

## 3. Results 

### 3.1. CD44^high^/CD24^low^ MCF-7 Cells Were More Resistant to Paclitaxel than Their Parental Cells

**Figure 1:**
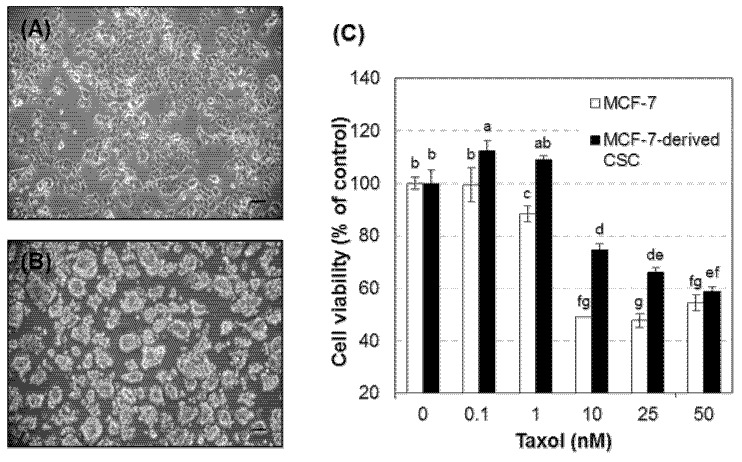

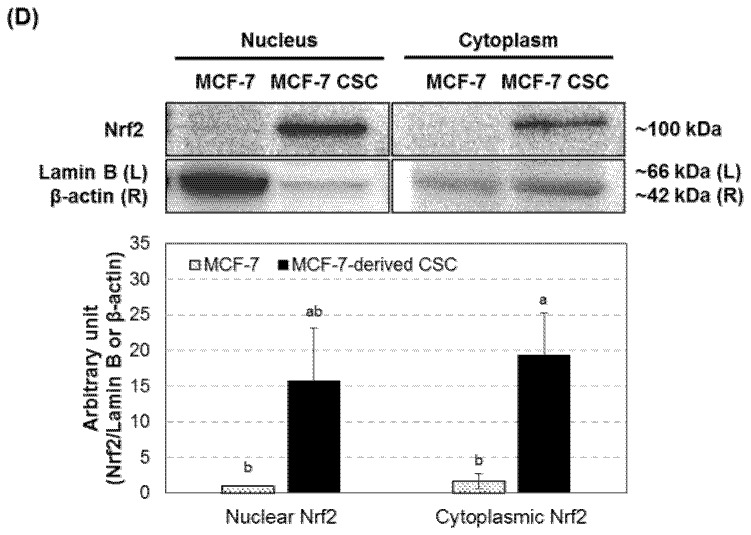
MCF-7-derived CSCs were more resistant to paclitaxel with higher expression of Nrf2 than MCF-7 cells. (**A**,**B**) Representative phase contrast images of MCF-7 cells (**A**) and MCF-7-derived CSCs (**B**) in culture. Scale bars in the images indicate 100 μm. (**C**) Cell viability in the presence of various concentrations of Taxol (1, 0.1, 1, 10, 25, and 50 nM). (**D**) Nrf2 protein expression levels in nuclear and cytoplasmic fractions from MCF-7 cell and MCF-7-derived CSC lysates. Representative immunoblot image (upper) and quantitative data (lower). *N* (independent experimental sessions) = 4; error bars, mean ± SEM. Values not sharing common letter on bar indicate statistically significant difference from each other (*p* < 0.05).

Breast CSCs were isolated from the adherent MCF-7 cultures by enriching a subpopulation immunoreactive for CD44 but rarely for CD24 [4,6,7,8]. The CSCs were grown in the maintenance medium as free-floating spherical aggregates that were different from the discrete and adherent MCF-7 cells in culture (Figure 1A,B). The cells were also able to re-grow in the sphere formation, which is consistent with the morphological characteristics that have been reported in various CSCs [29,30,31]. In addition, MCF-7-derived CSCs were more viable than their unsorted counterpart cells when treated with paclitaxel, one of the most commonly used anticancer drugs, at the concentrations deleterious to approximately 50% of MCF-7 cells (Figure 1C). The chemoresistance of these CSCs is consistent with the cellular feature widely reported for the breast CSCs [32,33]. Furthermore, we found that Nrf2 proteins are highly expressed in both the cytoplasmic and nuclear compartments of MCF-7-derived CSCs compared to their parental MCF-7 cells (Figure 1D). 

### 3.2. Chestnut Leaf Extract Suppressed ARE-Luciferase Activity

**Figure 2:**
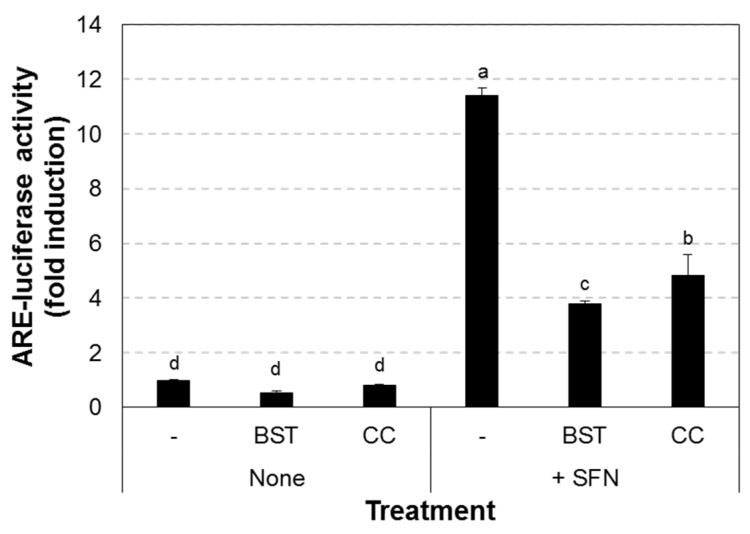
Chestnut leaf extract reduced antioxidant response element (ARE)-luciferase activity. HepG2-ARE cells were plated in a 6-well culture plate and treated with the indicated agents for 4 h. CC, *Castanea crenata* leaf extract, 50 μg/mL; SFN, sulforaphane, an ARE activator, 5 μM; BST, Brusatol, an Nrf2 inhibitor, 40 nM. *N* = 3; error bars, mean ± SEM. Values not sharing common letter on bar indicate statistically significant difference from each other (*p* < 0.05).

**Figure 3:**
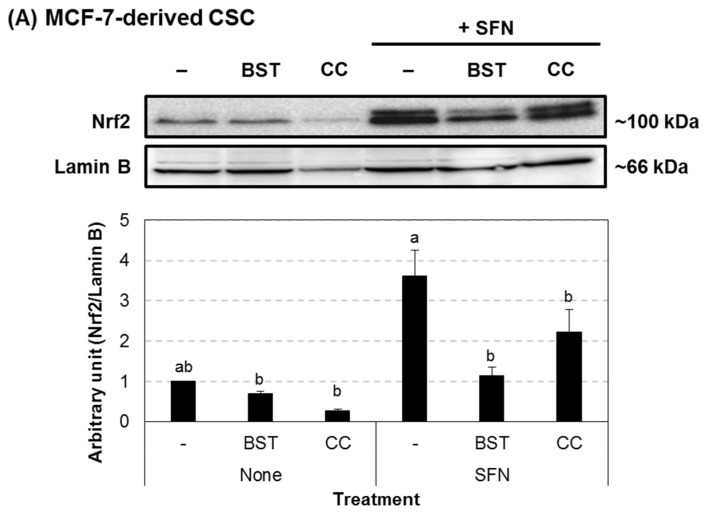

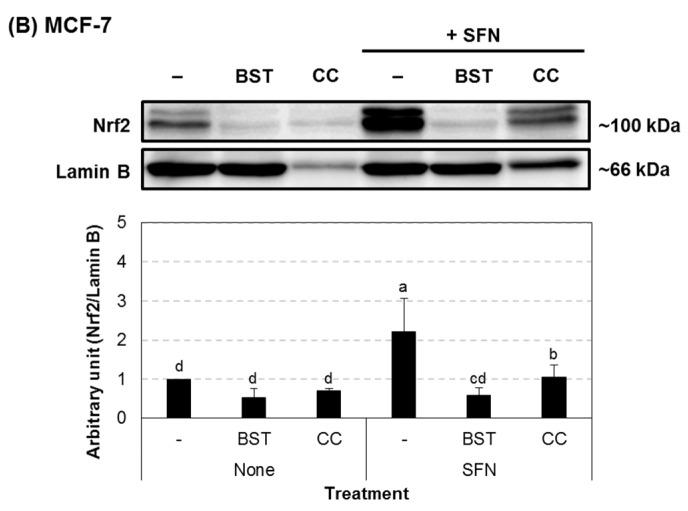
Chestnut leaf extract suppressed nuclear translocation of Nrf2 protein in MCF-7-derived CSCs and their counterparts. Both MCF-7-derived CSCs (**A**) and their parental cells (**B**) were treated with either chestnut leaf extract (CC, 50 μg/mL) or an Nrf2 inhibitor, brusatol (BST, 40 nM) in the absence or presence of an Nrf2 activator, sulforaphane (SFN, 5 μM). Representative immunoblot image (upper) and quantitative data (lower). *N* = 3; error bars, mean ± SEM. Values not sharing common letter on bar indicate statistically significant difference from each other (*p* < 0.05).

**Figure 4:**
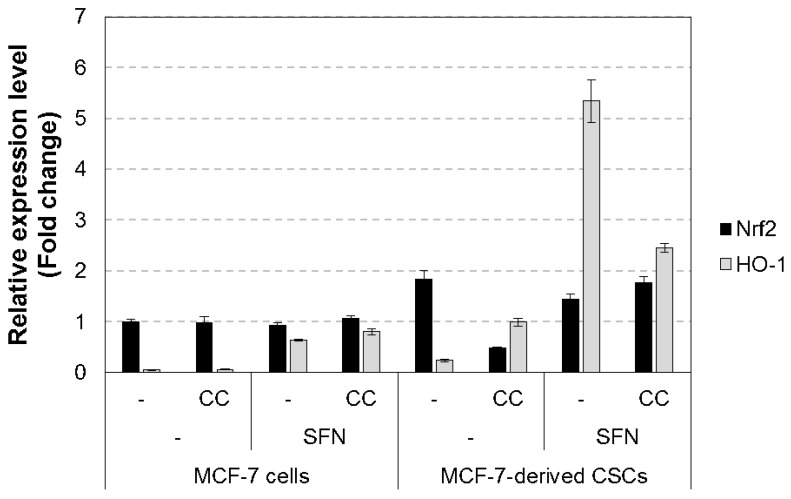
Chestnut leaf extract suppressed the mRNA levels of Nrf2 and its downstream genes in MCF-7-derived CSCs. Relative mRNA levels for indicated genes in MCF-7-derived CSCs were quantified using qPCR. The NCBI accession number for each gene and the information of primer sets are shown in Table 1. Nrf2, NF-E2-related factor 2; HO-1, heme oxygenase 1. *N* = 3; error bars, mean ± SEM.

A total of 89 plant extracts were screened for their ability to inhibit the transcriptional activity of Nrf2 using the ARE-luciferase assay in HepG2-ARE cells (Supplementary Figure S1). Among them, *Castanea crenata* (chestnut) leaf extract at 50 μg/mL, which was non-cytotoxic (Supplementary Figure S2), significantly inhibited ARE-luciferase activity, and its activity was comparable to brusatol, a known Nrf2 inhibitor (Figure 2). We also found that the extract reduced Nrf2 protein levels in nuclear fractions of both MCF-7-derived CSCs and their parental cells while nuclear Nrf2 levels were increased after sulforaphane treatment (Figure 3). Moreover, Nrf2 transcript level was prominently decreased in the extract-treated CSCs (Figure 4). In addition, the expression of heme oxygenase 1 (HO-1), a gene downstream of Nrf2, was increased by sulforaphane [27,28], whereas it was attenuated after treatment with the chestnut leaf extract. 

### 3.3. Chestnut Leaf Extract Increased Chemosensitivity of CSCs

**Figure 5:**
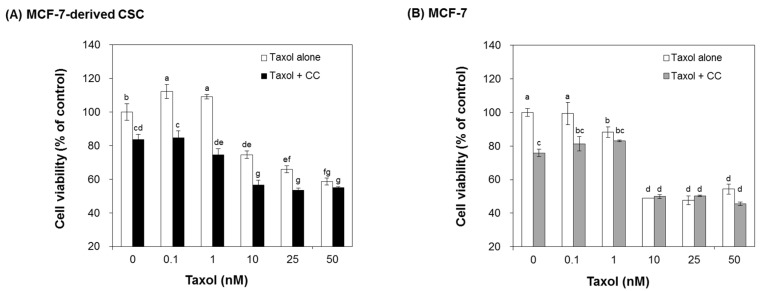
Chestnut leaf extract-treated CSCs were more susceptible to Taxol treatment than untreated CSCs. Both MCF-7-derived CSCs (**A**) and their parental cells (**B**) were treated with chestnut leaf extract in the presence of Taxol at various concentrations (0, 0.1, 1, 10, 25, and 50 nM). The CSCs exposed to the extract became more vulnerable to Taxol at ≥1 nM, compared to the cells that were not exposed to the extract. CC, *Castanea crenata* leaf extract, 50 μg/mL. *N* = 3; error bars, mean ± SEM. Values not sharing common letter on bar indicate statistically significant difference from each other (*p* < 0.05).

To examine whether chestnut leaf extract causes the sensitivity of CSCs to paclitaxel, MCF-7-derived CSCs and their parental cells were cultured in the presence of chestnut leaf extract and various concentrations of paclitaxel. The cell viability was assayed after 48 h (Figure 5). Treatment with chestnut leaf extract rarely influenced both cell types in the presence of paclitaxel at ≤0.1 nM, compared to the cells cultured with the extract but without paclitaxel. Interestingly, co-treatment with the extract and paclitaxel at ≥1 nM significantly reduced the viability of CSCs, compared to the treatment with paclitaxel alone but did not have any significant effect on the viability of MCF-7.

### 3.4. Chestnut Leaf Extract Facilitated Paclitaxel-Induced Apoptotic Cell Death

**Figure 6:**
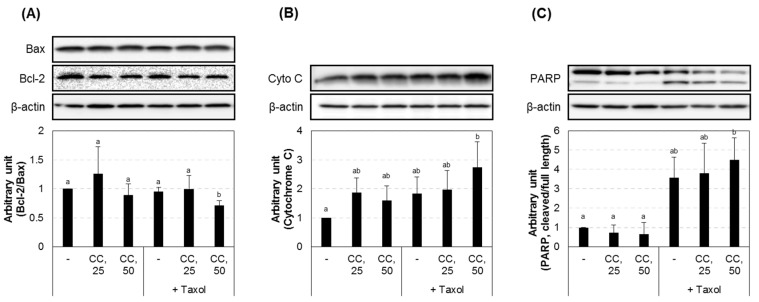
Chestnut leaf extract promoted the cytoplasmic levels of apoptosis-associated proteins in MCF-7-derived CSCs. (**A**–**C**) Immunoblot images and quantified data for Bax and Bcl-2 (**A**,**B**), cytochrome C (**B**), and cleaved PARP (**C**). Taxol, paclitaxel (10 nM); CC, *Castanea crenata* leaf extract (25 or 50 μg/mL). *N* = 3; error bars, mean ± SEM. Values not sharing common letter on bar indicate statistically significant difference from each other (*p* < 0.05).

Apoptosis-associated protein expression levels were further examined in CSCs treated with paclitaxel in the absence or presence of the extract (Figure 6). Co-treatment with the extract and paclitaxel decreased the expression ratio of Bcl-2 to Bax and increased the level of cytochrome C and cleaved PARP compared to the untreated cells or cells treated with paclitaxel alone. That is, chestnut leaf extract promoted the cytoplasmic levels of apoptosis-associated proteins in MCF-7-derived CSCs and their parental cells, which was consistent with the observation from JC-1 staining (Supplementary Figure S3). Chestnut leaf extract facilitated paclitaxel-induced mitochondrial damage, a hallmark for apoptotic cell death. In MCF-7-derived CSCs and their parental cells treated with paclitaxel and the extract for 12 h, the ratio of green fluorescent JC-1 monomers to red fluorescent J-aggregates was further increased, compared to the cells treated with paclitaxel alone. This suggests that chestnut leaf extract may further potentiate paclitaxel-induced mitochondrial membrane depolarization.

### 3.5. Chestnut Leaf Extract Impeded Colony Formation of CSCs

**Figure 7:**
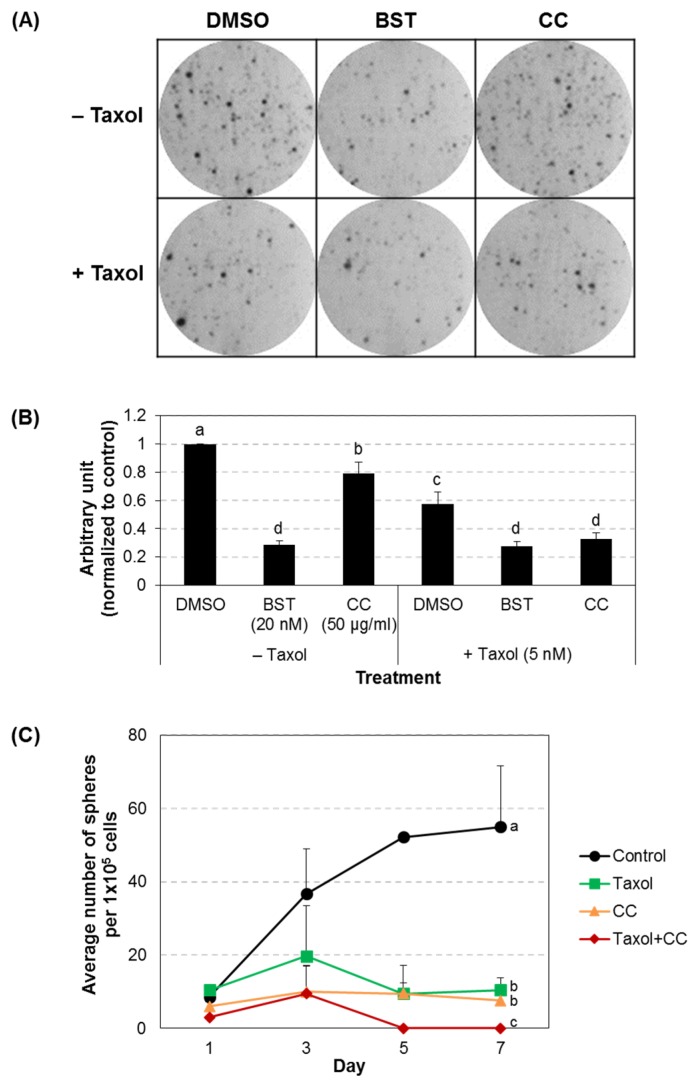
Chestnut leaf extract inhibited colony formation. (**A**,**B**) MCF-7-derived CSCs were cultured in a 6-well plate coated with matrigel, treated with the designated agents, and analyzed using crystal violet-based clonogenic assay. (**A**) Representative images. (**B**) Quantified data. (**C**) Sphere-forming ability of MCF-7-derived CSCs. After sorting for CSC phenotype, 1 × 10^5^ cells CD44^high^/CD24^low^ MCF-7 cells were cultured in a well of a 24-well culture plate. Cells were grown in CSC maintenance medium with the designated treatment for 7 days. Free-floating spherical aggregates formed in a well were counted every other day. Taxol, paclitaxel (5 nM); BST, Brusatol (20 nM); CC, *Castanea crenata* leaf extract (50 μg/mL). *N* = 3; error bars, mean ± SD. Values not sharing common letter on bar indicate statistically significant difference from each other (*p* < 0.05).

Breast CSCs are known to possess clonogenic ability and form mammospheres [4]. To determine whether chestnut leaf extract influences the clonogenic activity of MCF-7-derived CSCs under colony forming culture conditions, the cells were plated in a 6-well plate coated with matrigel, treated with the extract in the absence or presence of paclitaxel for 5 days, and analyzed via crystal violet-based clonogenic assay (Figure 7A,B). Quantified data showed that either paclitaxel or chestnut leaf extract significantly inhibited colony formation of CSCs cultured on a matrigel substrate, as the known Nrf2 inhibitor brusatol did [23]. In addition, colony formation was further suppressed by a combined treatment with paclitaxel and the extract. This observation was consistent with the result from CSC suspension cultures in the maintenance medium (Figure 7C). These findings suggest that chestnut leaf extract hindered colonic growth of CSCs in two- or three-dimensional circumstances and that combination of the extract and paclitaxel inhibited clonal expansion of CSC more effectively than either treatment alone.

## 4. Discussion and Conclusions

In an effort to improve adjuvant chemotherapy efficacy, we screened various edible plant extracts for an inhibitory effect against Nrf2-mediated antioxidant responses and found that chestnut leaf extract was most effective in inhibiting Nrf2 signaling pathway whose upregulation was associated with reduced efficacy of anticancer drugs including paclitaxel. Our results demonstrated that the CSCs constitutively expressed Nrf2 at a relatively high level and exhibited less sensitivity to paclitaxel than the parental MCF-7 cells. As expected, chestnut leaf extract suppressed Nrf2 signaling pathway as assessed by ARE-luciferase activity, further promoted paclitaxel-induced apoptosis, and inhibited the colony forming ability of CSCs. It is highly plausible that chestnut leaf extract or its constituents sensitized MCF-7-derived CSCs to paclitaxel by suppressing Nrf2-mediated intracellular antioxidant systems and increasing intracellular ROS level, and thereby induced apoptotic cell death. 

CSCs are responsible for tumor development—initial formation, metastasis, and relapse, as well as drug-resistance; thus, CSCs are emerging as a target in the development of effective cancer therapeutic strategies [1]. Within CSCs, the levels of ROS, highly reactive byproducts of aerobic metabolism, are maintained relatively low so as to produce less DNA damage [5,34]. Lower intracellular ROS levels in CSCs than in cancer non-stem cells are reportedly due to the activated radical scavenging machinery, by the activities of highly expressed antioxidant enzymes such as superoxide dismutase 1 (SOD1) and glutathione synthase [5]. That is, the enhanced antioxidant defense system in CSCs contributes to their tumorigenicity and resistance to radio- or chemo-therapy [5,14] although it remains unclear how the redox balance is intrinsically controlled in the CSCs [35]. Therefore, the eradication of CSCs could be substantialized by regulating ROS levels and manipulating their antioxidant capacity. 

A master regulator of antioxidant and detoxification responses, Nrf2, is known to be activated in different types of CSC models, including breast CSCs [4,16]. The activated Nrf2 signaling pathway and decreased ROS levels are closely correlated with increased clonogenicity and chemoresistance of CSCs [16]. Multiple studies have shown that naturally occurring compounds such as brusatol [23], apigenin [36], luteolin [37], and trigonelline [38] could interfere with Nrf2 signaling in several types of cancer cells [39]. However, their repressive modes of action have not yet been characterized [23]. Recently, brusatol was found to inhibit general translation of many short-lived proteins, rather than to target Nrf2 in a direct and specific manner [40]. Our results demonstrated that chestnut leaf extract slightly decreased the levels of Nrf2 transcripts and proteins, compared to the control, in the CSCs not treated with SFN, which is suggestive for a transcriptional and translational inhibition of Nrf2 expression by chestnut leaf extract. However, further studies are needed to identify the molecular mechanism though which the extract suppresses the function of Nrf2 in CSCs.

The MCF-7-derived CSCs that were generated and examined in this study have shown a notable abundance in Nrf2 mRNA transcripts and proteins with greater clonogenic capability and chemoresistance to paclitaxel in comparison to the adherent MCF-7 cells. In addition, chestnut leaf extract treatment was found to inhibit activation of the ARE-Nrf2 antioxidant defense axis of MCF-7-derived CSCs, suppressed cell growth in the form of sphere, and enhanced cell susceptibility to paclitaxel. It suggests that the chestnut leaf extract may have potential to inhibit Nrf2 signaling pathway by repressing both nuclear translocation and transcription of Nrf2, leading to sensitization of breast CSCs to paclitaxel. However, further systematic and molecular studies are required to understand intracellular dynamics in the sensitized CSCs.

Chestnuts belong to the genus *Castanea* in the family Fagaceae; *C. crenata*, commonly known as Korean or Japanese chestnut, is one of four main species, including European chestnut (*C. sativa*), American chestnut (*C. dentate*), and Chinese chestnut (*C. mollissima*) [41]. It has been reported that *C. crenata* extract has an anticancer effect by inhibiting cancer cell proliferation and promoting apoptosis when tested in a gastric cancer cell line [42]. The extracts from its inner and outer shell and leaves have antioxidant activity to scavenge free radicals [43,44,45]. In the corresponding context, the extracts of *C. sativa* leaves in ethyl acetate, methanol or water highly contain phenolic compounds and possess strong radical scavenging potential, which contributes to their biological benefits to in vitro and in vivo model systems by ameliorating the oxidative stress-induced detrimental effects [46,47]. A study by Quave et al. demonstrated that ethyl acetate fraction of methanol extract from *C. sativa* leaves richly contains ursene and oleanene-type triterpenes [48]. Calabria et al. have reported that ursene and oleanene-type triterpenes isolated from botanical sources had a cytotoxic effect in a human breast cancer cell line, MDA-MB-231 [49]. Consistent with these findings, our unpublished data showed that Nrf2-inhibiting potential of *C. crenata* methanol extract was maximized in ethyl acetate fraction among solvent fractions including n-hexane, methylene chloride, ethyl acetate, n-butanol, and water. Therefore, it is reasonably speculated that *C. crenata* leaf extract may contain bioactive chemical components feasibly fractionated in ethyl acetate such as pentacyclic triterpenes. Detailed physicochemical characterization of bioactive constituents in chestnut leaf extract awaits further study.

Paclitaxel is a microtubule-stabilizing drug that causes cancer cell death by generating and accumulating intracellular ROS [50,51]. Moreover, cellular antioxidant capacity is proportionally correlated with paclitaxel resistance [52]. Thus, it is conceivable that chestnut leaf extract, which could suppress the Nrf2-mediated antioxidant system, may potentiate ROS accumulation and consequently facilitate paclitaxel-induced apoptotic cell death. Indeed, our results showed that chestnut leaf extract treatment decreased the expression ratio of Bcl-2/Bax, increased the level of cleaved PARP, and stimulated mitochondrial damage in paclitaxel-treated CSCs compared to untreated or extract alone-treated cells. This suggests that the combined usage of chestnut leaf extract with paclitaxel could efficiently achieve paclitaxel-induced CSC elimination. Therefore, synergistic effects are expected when the chestnut leaf extract and paclitaxel are used in combination [53]; however, rigorous evaluation through further in vitro and in vivo studies are required to examine potential adverse effects by combined usage—for example, counteraction and/or additive toxicity of the two agents [54].

Taken together, we successfully isolated and cultivated MCF-7-derived CSCs possessing CD44^high^/CD24^low^ phenotype, mammospheres-forming capability, and paclitaxel-resistance, from their adherent parental cells. Also, methanol extract of chestnut leaf was found to inhibit the function of a master regulator of cellular antioxidant responses, Nrf2, and attenuated paclitaxel-resistance of the CSCs. These findings suggest that chestnut extract or its constituents could be exploited in order to increase the therapeutic efficacy of conventional anticancer drugs. 

## Figures and Tables

**Table 1:** Primer sets for real-time PCR.

Gene (NCBI Accession No.)	Primer (5′→3′)		Product Length (bp)
	Forward	Reverse	
*Nrf2* (NM_006164)	CATCCAGTCAGAAACCAGTGG	GCAGTCATCAAAGTACAAAGCAT	85
*Keap1* (NM_012289)	CAGATTGGCTGTGTGGAGTT	GCTGTTCGCAGTCGTACTTG	202
*HO-1* (NM_002133)	TCCTGGCTCAGCCTCAAATG	CGTTAAACACCTCCCTCCCC	107
*GAPDH* (NM_001289746)	ACCCACTCCTCCACCTTTGA	CTGTTGCTGTAGCCAAATTCGT	101

NCBI, National Center for Biotechnology Information; Nrf2, NF-E2-related factor 2; Keap1, Kelch-like ECH-associated protein 1; HO-1, heme oxygenase 1; GAPDH, glyceraldehyde 3-phosphate dehydrogenase.

## Supplementary Materials

The following are available online at http://www.mdpi.com/2072-6643/9/7/760/s1, Figure S1: Various plant extract samples influencing ARE-luciferase activity, Figure S2: Cytotoxicity of chestnut leaf extract. MCF-7 cells were treated with the extract at various concentrations (0, 6.25, 12.5, 25, 50, 100, and 200 μg/mL), Figure S3: Chestnut leaf extract facilitated paclitaxel-induced mitochondrial damage, a hallmark for apoptotic cell death.
