# Supplementary material for: Suppression of Nrf2 Activity by Chestnut Leaf Extract Increases Chemosensitivity of Breast Cancer Stem Cells to Paclitaxel
Source: Nutrients. 2017 Jul 18;9(7):760. doi: 10.3390/nu9070760 (PMC5537874; doi:10.3390/nu9070760)
Supplement: Supplementary file 1 [file nutrients-09-00760-s001.zip › nutrients-200549-supplementary.pdf]

Supplementary Figures

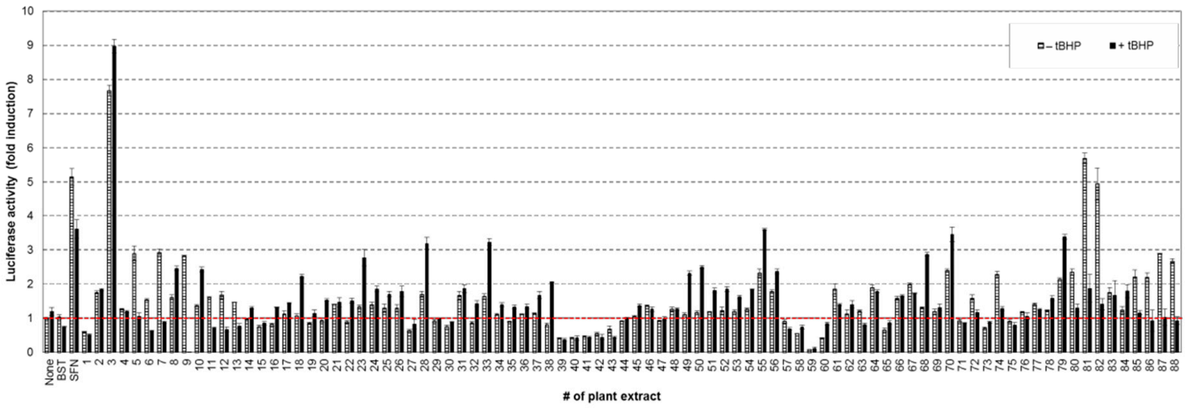

Supplementary Figure S1. Various plant extract samples influencing ARE-luciferase activity. HepG2-ARE cells were treated with the extracts (50 µg/mL), and luciferase activity in the absence or presence of each extract was analyzed. SFN, sulforaphane (5 µM); BST, Brusatol (40 nM). N = 3; error bars, mean ± SEM.

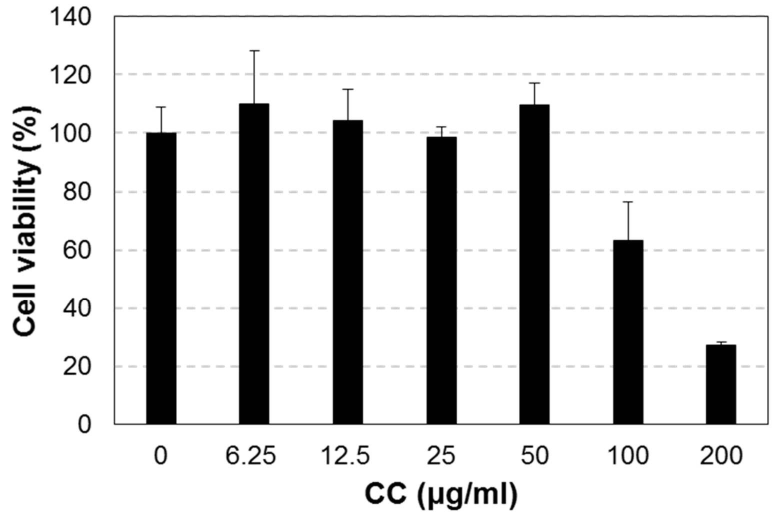

Mean (%)	100.00	109.91	104.31	98.71	109.77	63.36	27.16
SD	8.94	18.08	10.57	3.45	7.31	13.21	1.02

Supplementary Figure S2. Cytotoxicity of chestnut leaf extract. MCF-7 cells were treated with the extract at various concentrations (0, 6.25, 12.5, 25, 50, 100, and 200 μg/mL). After 24-h incubation under starvation conditions (0.5% FBS-containing maintenance medium), cell viability was assayed using CCK-8 kit. CC, *Castanea crenata* leaf extract. N = 2; error bars, mean ± SD.

(A)

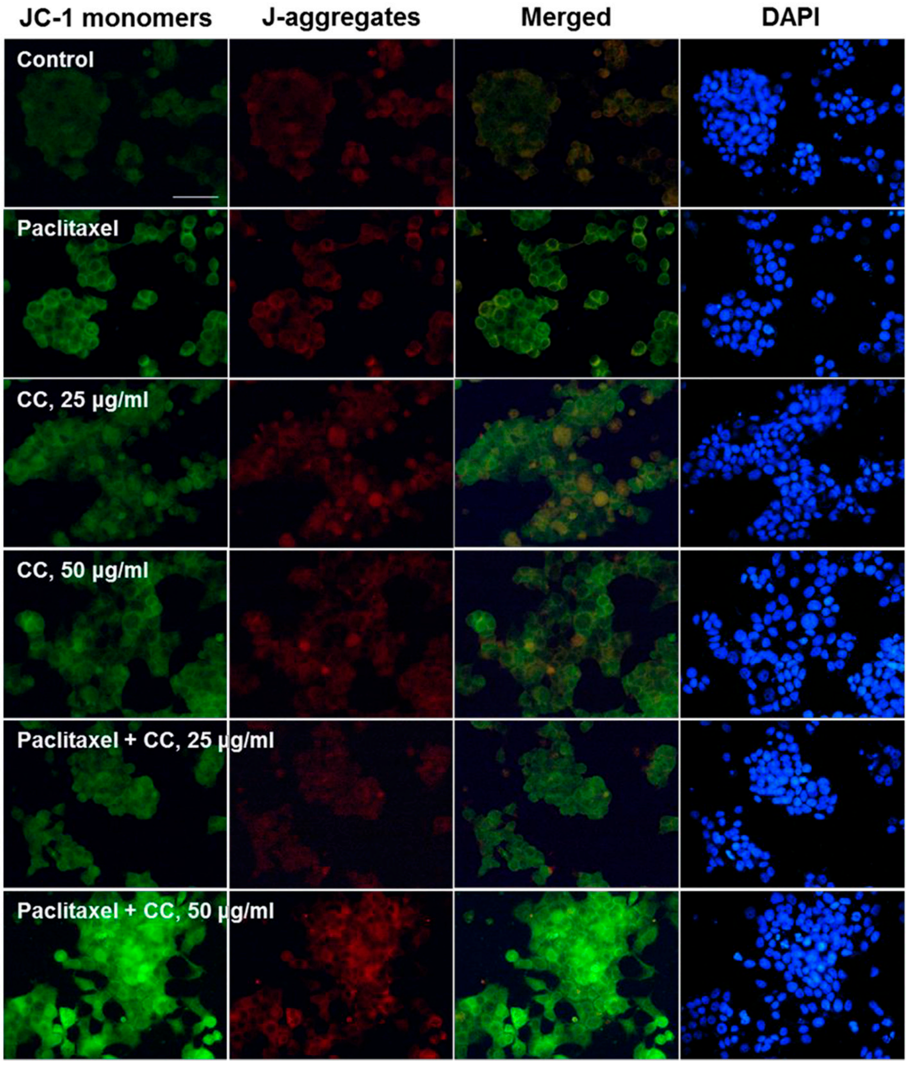
JC-1 monomers
J-aggregates
Merged
DAPI
Control
Paclitaxel
CC, 25 µg/ml
CC, 50 µg/ml
Paclitaxel + CC, 25 µg/ml
Paclitaxel + CC, 50 µg/ml

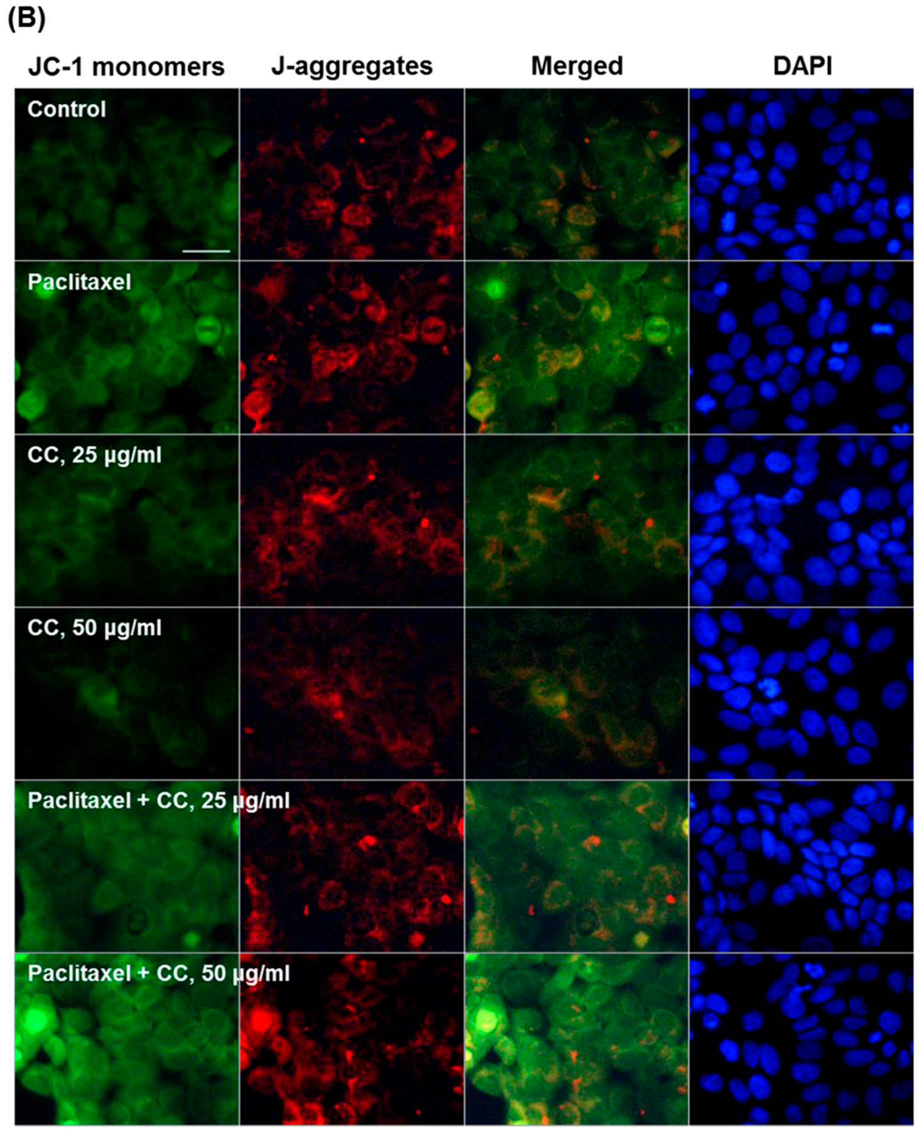

Supplementary Figure S3. Chestnut leaf extract facilitated paclitaxel-induced mitochondrial damage, a hallmark for apoptotic cell death. (A-B) MCF-7-derived CSCs (A) and their parental MCF-7 cells (B) were treated with paclitaxel with or without the extract for 12 h. The mitochondrial membrane depolarization was assessed via JC-1 staining. Green fluorescent JC-1 monomers produce red fluorescent J-aggregates within the mitochondria of healthy cells, whereas the monomers remain in the cytoplasm of apoptotic cells that have undergone mitochondrial membrane depolarization. Scale bars in (A) and (B) indicate 100 µm and 50 µm, respectively.
